# Human Subperitoneal Fibroblast and Cancer Cell Interaction Creates Microenvironment That Enhances Tumor Progression and Metastasis

**DOI:** 10.1371/journal.pone.0088018

**Published:** 2014-02-04

**Authors:** Motohiro Kojima, Youichi Higuchi, Mitsuru Yokota, Genichiro Ishii, Norio Saito, Kazuhiko Aoyagi, Hiroki Sasaki, Atsushi Ochiai

**Affiliations:** 1 Pathology Division, Research Center for Innovative Oncology, National Cancer Center Hospital East, Kashiwa, Chiba, Japan; 2 Colorectal and Pelvic Surgery Division, National Cancer Center Hospital East, Kashiwa, Chiba, Japan; 3 Genetic Division, National Cancer Center Research Institute, Chuo-ku, Tokyo, Japan; Hirosaki University Graduate School of Medicine, Japan

## Abstract

**Backgrounds:**

Peritoneal invasion in colon cancer is an important prognostic factor. Peritoneal invasion can be objectively identified as periotoneal elastic laminal invasion (ELI) by using elastica stain, and the cancer microenvironment formed by the peritoneal invasion (CMPI) can also be observed. Cases with ELI more frequently show distant metastasis and recurrence. Therefore, CMPI may represent a particular milieu that facilitates tumor progression. Pathological and biological investigations into CMPI may shed light on this possibly distinctive cancer microenvironment.

**Methods:**

We analyzed area-specific tissue microarrays to determine the pathological features of CMPI, and propagated subperitoneal fibroblasts (SPFs) and submucosal fibroblasts (SMFs) from human colonic tissue. Biological characteristics and results of gene expression profile analyses were compared to better understand the peritoneal invasion of colon cancer and how this may form a special microenvironment through the interaction with SPFs. Mouse xenograft tumors, derived by co-injection of cancer cells with either SPFs or SMFs, were established to evaluate their active role on tumor progression and metastasis.

**Results:**

We found that fibrosis with alpha smooth muscle actin (α-SMA) expression was a significant pathological feature of CMPI. The differences in proliferation and gene expression profile analyses suggested SPFs and SMFs were distinct populations, and that SPFs were characterized by a higher expressions of extracellular matrix (ECM)-associated genes. Furthermore, compared with SMFs, SPFs showed more variable alteration in gene expressions after cancer-cell-conditioned medium stimulation. Gene ontology analysis revealed that SPFs-specific upregulated genes were enriched by actin-binding or contractile-associated genes including α-SMA encoding ACTA2. Mouse xenograft tumors derived by co-injection of cancer cells with SPFs showed enhancement of tumor growth, metastasis, and capacity for tumor formation compared to those derived from co-injection with cancer cells and SMFs.

**Conclusions:**

CMPI is a special microenvironment, and interaction of SPFs and cancer cells within CMPI promote tumor growth and metastasis.

## Introduction

Although tumor size is a major prognostic factor in many cancers, prognosis in gastrointestinal cancer is stratified not by tumor size but by tumor spread [1]. Peritoneal invasion in colorectal cancer has been reported to be a strong prognostic factor, but this term was not well defined, and detection and diagnosis methods have been questioned [2]–[4]. Recent pathological reports have demonstrated that elastica stain, which highlights the peritoneal elastic lamina near the periotoneal surface, is useful for objective detection of peritoneal invasion. We and others have determined that peritoneal invasion defined as tumor invasion beyond the peritoneal elastic lamina (elastic laminal invasion: ELI) is a strong prognostic factor that can influence future pT criteria in the Union for International Cancer Control (UICC) TNM classification [5]–[7]. The peritoneum is a very thin membrane, within 500 µm thick, and the peritoneal elastic lamina exists within this membrane. The frequency of synchronous metastasis and recurrence is increased by 2 to 4 times when a tumor invades this narrow space [5]. These results may suggest that tumor progression and metastasis are facilitated by a cancer microenvironment formed by peritoneal invasion (CMPI). The extent of CMPI can be identified by using elastica stain, and pathological features of CMPI can also be determined.

A tissue microarray facilitates the evaluation of protein expression for a large number of tissue blocks from a single specimen, and area-specific tissue microarrays have been reported to be useful for studying specific tumor areas in large cohorts [8]. After determination of CMPI by using elastica stain, a tissue core can be obtained from this area and a comparison with the features of other tumor areas can also be performed. This process may allow for an assessment of the important biological phenomena occurring in this cancer microenvironment.

Recent advances in cancer research have established the concept of cancer microenvironment that promotes tumor initiation, invasion, and metastasis [9]. Although the cancer microenvironment is composed of many types of cells, the use of area-specific tissue microarrays may allow for a focus on the cell components that characterize CMPI. Furthermore, if these cell components can be cultivated from the histologically corresponding subperitoneal region, a biological study to elucidate this putative cancer-promoting microenvironment can be performed.

The aim of this study was to explain how the colorectal cancer prognosis is affected by peritoneal invasion. We constructed area-specific tissue microarray system to determine the characteristic cell components of CMPI. Next, we cultivated specific fibroblast subpopulations from the submucosal and subperitoneal layers of the human colonic wall. The biological characteristics and gene profiles of submucosal fibroblasts (SMFs) and subperitoneal fibroblasts (SPFs) with or without cancer-cell-conditioned medium (CCCM) stimulation were compared. Subsequently, we constructed xenograft tumors by co-injection of cancer cells with either SPFs or SMFs. Our study proposed a new candidate for a cancer-promoting microenvironment in colon cancer and elucidated SPFs as crucial players in the enhancement of tumor progression and metastasis.

## Patients and Methods

### Ethics Statement

This study was approved by the National Cancer Center Hospital East Institutional Review Board (No: 19-021). A written general consent to use biologic materials for research was obtained from each participant prior to tissue acquisition. Animal experiments were approved by the Animal Ethics Committee of the National Cancer Center Hospital East (K11-032).

### Patient Characteristics and Detection of ELI

Four hundred consecutive patients with TNM classification (5^th^ edition) pT3 and pT4a colon cancer [10], undergoing surgery between 1996 and 2003 at the National Cancer Center Hospital East, were enrolled. Using elastica stain, we identified 173 cases with ELI and further examined these using area-specific tissue microarrays [5],[8]. Of the 173 cases with ELI, 107 were pT3 and 66 were pT4a.

### Construction of Area-Specific Tissue Microarrays

**Figure 1:**
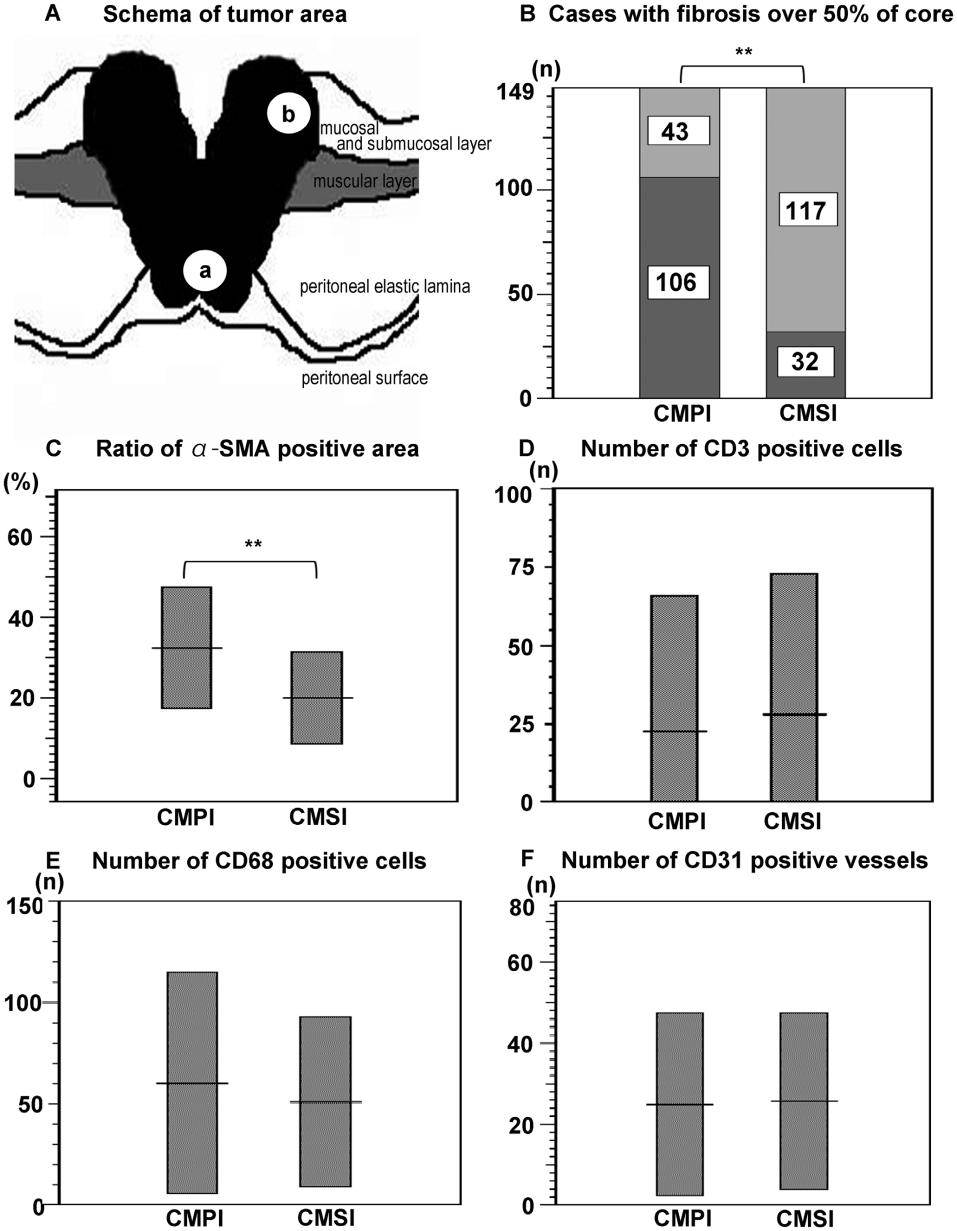
Pathological features of tumor microenvironment explored by using area-specific tissue microarray. (A) Schema of the cancer microenvironment formed by peritoneal invasion (CMPI) and the cancer microenvironment formed by submucosal invasion (CMSI) defined as (a) invasive front with peritoneal invasion and (b) submucosal invasive front, respectively. (B) The distribution of fibrosis in human colon cancer tissue. Dark gray bars show the number of the cases with fibrosis over 50% of the core from each tumor area, and light gray bars show the number of the cases without extensive fibrosis. Core samples with CMPI showed a higher frequency of marked fibrosis than did core samples with CMSI (*P*<0.01). (C) Distribution of α-SMA expression in human colon cancer tissue. CMPI showed higher α-SMA expressions than those seen in CMSI (*P*<0.01). (D) Distribution of CD3-positive cells in human colon cancer tissue. Numbers of CD3-positive cells were not significantly different between CMPI and CMSI. (E) Distribution of CD68-positive cells in human colon cancer tissue. Numbers of CD68-positive cells were not significantly different between CMPI and CMSI. (F) Distribution of CD31-positive vessels in human colon cancer tissue. Numbers of the CD31-positive vessels were not significantly different between CMPI and CMSI. Results in (B) are presented by case numbers, and those in (C–F) are presented as the mean ± SD of 149 cases (**P<0.01).

To elucidate the pathological features of CMPI in colon cancer tissue, we defined the cancer microenvironment as follows: (a) CMPI has a tumor border with peritoneal invasion and (b) the cancer microenvironment formed by submucosal invasion (CMSI) has a submucosal invasive tumor border (Figure 1A) [5]. The 2-point tissue microarray was then established as previously described [8]. Each tumor area was marked with ink on the histological slide; a single tissue core of 2 mm in diameter was obtained from each cancer microenvironment and transferred to a recipient block using a Tissue Microarrayer (Azumaya, Tokyo, Japan). In 24 cases, insufficient cancer tissue was obtained from the CMPI. However, sufficient tissue was obtained in 149 cases; these were analyzed histologically and immunohistochemically (See Materials and Methods S1, and Table S1).

### Antibodies, Regents, and Immunohistochemistry

The antibodies, reagents, and the immunohistochemical procedures used are described in Materials and Methods S1 and the Table S2.

### Evaluation of Area-Specific Tissue Microarray Sections

High-resolution slide images were acquired from all tissue cores with hematoxylin-eosin (H.E) and immunohistochemistry staining, using NanoZoomer 2.0-HT slide scanner (Hamamatsu photonics, Hamamatsu, Japan). All cores were examined using viewer software (NDP view: Hamamatsu photonics, Hamamatsu, Japan). When the area of fibrosis exceeded 50% of a whole tissue core with a 2 mm diameter upon H.E staining, it was defined as positive for marked fibrosis. On immunohistochemical staining, hot spots with CD3-, CD31-, and CD68-positive cells or vessels were selected in the viewer software, then an image of x20 magnification (0.51 mm^2^) was taken, and saved as a JPEG file. Positive cells or vessels were counted in each image using morphometric software (WinRoof, Mitani Corporation, Fukui, Japan). Moreover, the area with highest alpha smooth muscle actin (α-SMA) expression in fibroblasts was selected, then a x20 magnification (0.51 mm^2^) image was taken, and saved as a JPEG file. The ratio of the α-SMA positive area in the image was calculated using morphometric software, as described previously [11]. The α-SMA expression in normal muscle tissue, as determined by comparison with a serial H.E slide, was not evaluated. H.E and immunohistochemical staining data of CMPI was compared with that of CMSI to elucidate the histological characteristics of CMPI.

### Primary Cells and Cell Lines

Submucosal tissue was obtained from sigmoid colon tissue more than 5 cm distant from the tumor. Colonic tissue was dissected from the muscular layer on the luminal side, and lamina propria and mucosal layer tissues were obtained. Next, the lamina propria was scrubbed away to obtain submucosal tissue. Subperitoneal tissue was obtained from the sigmoid colon mesentery at more than 5 cm distant from the tumor by using operating tweezers and scissors. These tissues were washed with phosphate-buffered saline (PBS) and incubated in 5% trypsin for 20 minutes, 3 times. The supernatant was centrifuged, plated on a dish, and submucosal fibroblasts (SMFs) and subperitoneal fibroblasts (SPFs) were obtained and then grown and maintained in MF-medium (Toyobo, Tokyo, Japan) [12]. All experiments were performed on cells within 8 passages.

The human colorectal cancer cell lines DLD-1 and Caco-2 were obtained from the American Type Culture Collection and grown in Dulbecco’s modified Eagle medium (DMEM) (Sigma-Aldrich, Saint Louis, MO) containing 100 U/mL penicillin, 100 µg/mL streptomycin (Sigma-Aldrich, Saint Louis, MO), and 10% fetal bovine serum (FBS; Gibco, Palo Alto, CA).

### Cell Proliferation Assay, Immunocytochemical Staining, and Flow Cytometry Analysis

Cell proliferation assays, immunocytochemical staining, and flow cytometry analyses were performed as described in Materials and Methods S1.

### Stimulation of Fibroblasts by Cancer Cell Medium

Initially, 1.7×10^4^/cm^2^ of fibroblasts and DLD-1 cells were grown separately in DMEM containing 100 U/mL penicillin, 100 µg/mL streptomycin, and 10% FBS for 48 hours, and then were starved for 24 hours. Next, the medium was removed from the fibroblasts, and the medium from the starved DLD-1 cells was added to the fibroblasts for 24 hours to establish fibroblasts with cancer-cell-conditioned medium (CCCM) stimulation. As control, fibroblasts were starved for 48 hours (yielding fibroblasts without CCCM). SPFs and SMFs either with or without CCCM were assessed by using immunocytochemistry or gene expression analysis. As for the evaluation of immunocytochemical α-SMA expression, the area with highest α-SMA expression was selected, then a x20 magnification (0.51 mm^2^) image was taken, and saved as a JPEG file. The ratio of α-SMA positive area in the image was calculated using morphometric software (WinRoof, Mitani Corporation, Fukui, Japan).

### Gene Expression Analysis using Microarray

Three sets of SPFs and SMFs, either with or without CCCM, obtained from 3 different patients, were used in this study. We used GeneChip Human Genome U133 Plus 2.0 arrays (Affymetrix, Santa Clara, CA). Target cDNA was generated from 100 ng of total RNA extracted from each sample using a 3′ IVT Express Kit (Affymetrix, Santa Clara, CA). The procedures for target hybridization, washing and staining for signal amplification were performed according to the supplier’s protocols. The arrays were scanned with a Gene Chip Scanner 3000 (Affymetrix, Santa Clara, CA), and the intensity of each feature of the array was calculated by using GeneChip Operating Software, version 1.1.1 (Affymetrix, Santa Clara, CA). The average intensity was standardized to the target intensity, which was set equal to 1000, to reliably compare different arrays. The values were log transformed and median centered. The programs GeneSpring (Agilent Technologies, Santa Clara, CA) and Excel (Microsoft Corporation, Redmond, WA) were used to perform the numerical analyses for gene selection.

### Xenograft Transplantation and Tumor Formation Assay

Either 1×10^6^ human colorectal cancer cells Caco-2 or DLD-1 alone, or with either 1×10^6^ SPFs or SMFs, were injected subcutaneously (s.c.) into the back of SCID mice (8–12 weeks of age; CLEA, Tokyo, Japan). Tumor volumes were calculated weekly as described previously [13]. Mice injected with Caco-2 alone or with either SPFs or SMFs were killed after 10 weeks, and those injected with DLD-1 alone or with either SPFs or SMFs were killed after 8 weeks, and tumor weights were evaluated. For distant metastatic analysis, lung and liver tissue was removed and fixed in 10% formalin, and for the analysis of lymph node metastasis, neck and inguinal adipose tissue was also removed and fixed; all tissues were histologically examined. We used 8 mice in each group.

To elucidate the capacity of fibroblasts to enhance tumor formation, serial dilutions of Caco-2 or DLD-1 cancer cells were similarly co-injected with either 1×10^6^ SMFs or SPFs. Tumor formation was evaluated 4 weeks after the injection. We used 4 mice for each group.

### Statistical Analysis

*X* ^2^ test and Student’s *t* test were used in the tissue microarray analysis, cell proliferation assay, xenograft transplantation, and tumor formation assay. A *P*<0.05 was defined as statistically significant. In the microarray analysis, gene expression data were analyzed using GeneSpring GX12 (Agilent Technologies, Santa Clara, CA). Row data were summarized by using MAS5 and normalized by log transformation and median centering for numerical analyses for gene selection. For principal component analysis (PCA), we used probe sets that were reliably measured and varied by 3-fold above the global median in at least 2 samples (approximately 10%); analyses were performed using GeneSpring GX12. The differentially expressed probe sets used in supervised hierarchical clustering were selected based on *P*<0.05 and fold change (FC) >2.0. *P* values were calculated using one way ANOVA with Benjamini and Hochberg FDR multiple testing correction. Hierarchical clustering with weight-average linkage clustering was performed using Cluster and Treeview programs (Michael Eisen, Stanford University, genome-www.stanford.edu). The functional annotation clustering of Gene Ontology Enrichment analysis was performed using DAVID software, with the classification stringency set to “High”, and the significant clusters were selected based on an enrichment score >2.0 and a *P*<0.01 (Fisher’s exact test after Benjamini and Hochberg FDR multiple testing correction) [14],[15].

## Results

### Histological Features of ELI

**Figure 2:**
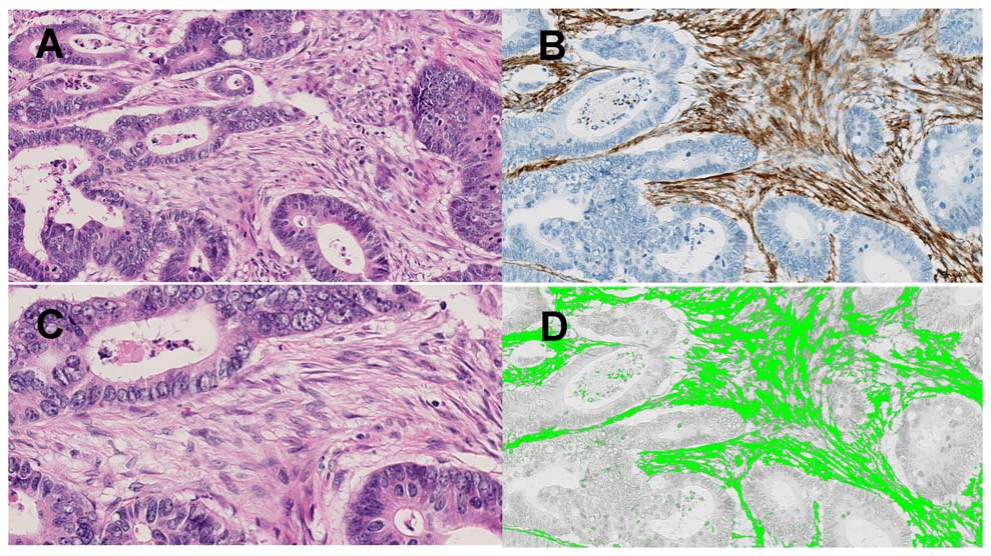
Histological features of fibrosis in the cancer microenvironment formed by peritoneal invasion (CMPI). (A) Histological features of stromal component of CMPI. Both in CMPI and the cancer microenvironment formed by submucosal invasion (CMSI), plump spindle-shaped fibroblasts were major sources of the stroma. (B) Marked α-SMA expression was found in fibroblasts. (C) Higher magnification more clearly revealed plump spindle-shaped fibroblasts. (D) Using morphometric software, we successfully detected and analyzed α-SMA expression.

Not only tumor cells, but also varieties of stromal cells constitute a distinct cancer microenvironment, and some promote tumor metastasis [9]. First, we elucidated the significant histological features of CMPI to shed light on phenomena occurring in this milieu by using area-specific tissue microarrays. The clinicopathological features of the 149 cases used are shown in Table S1. On H.E staining, we found extensive fibrosis (over 50%) more frequently in CMPI than was seen in CMSI (Figure 1B). The ratio of α-SMA positive area in CMPI was also higher than that seen in CMSI (Figure 1C). The proportions of T lymphocytes, macrophages, or microvessels evaluated using CD3, CD68, or CD31, respectively, were not significantly different between CMPI and CMSI (Figure 1D–F). Both in CMPI and CMSI, plump spindle-shaped fibroblasts were major source of α-SMA expression, and the ratio was successfully analyzed by using morphometric software (Figure 2A–D). Considering our previous results, which indicated that peritoneal invasion defined by ELI was closely associated with distant metastasis, we hypothesized that fibroblasts in the subperitoneal layer could be implicated not only in prominent fibrosis and activation, but also in the tumor’s progression and metastasis. We then decided to isolate fibroblasts from the subperitoneal layer that histologically corresponded to peritoneal invasion. Fibroblasts from the submucosal layer were used as controls.

### Isolation and Characterization of Cultured Human SPFs and SMFs

At first, we evaluated the morphological and biological characteristics of SPFs and SMFs in a normal state. Both cultured human SPFs and SMFs showed similar spindle-shaped morphologic characteristics (Figure S1A–B). SPFs and SMFs from 3 patients could be cultured over 10 passages, except for 1 SPF case (data not shown). Immunocytochemistry and flow cytometry revealed the obtained SPFs and SMFs were consistent with fibroblasts (Figure S1C–D). We found weak α-SMA expression in a few SPFs and SMFs. The doubling time for SPFs and SMFs was 79.9 hours and 36.3 hours, respectively, and the growth of SMFs was faster than that of SPFs (*P*<0.05), which suggested a biological difference between SPFs and SMFs (Figure S1E).

### Gene Expression Profiling Comparison between SPFs and SMFs

**Figure 3:**
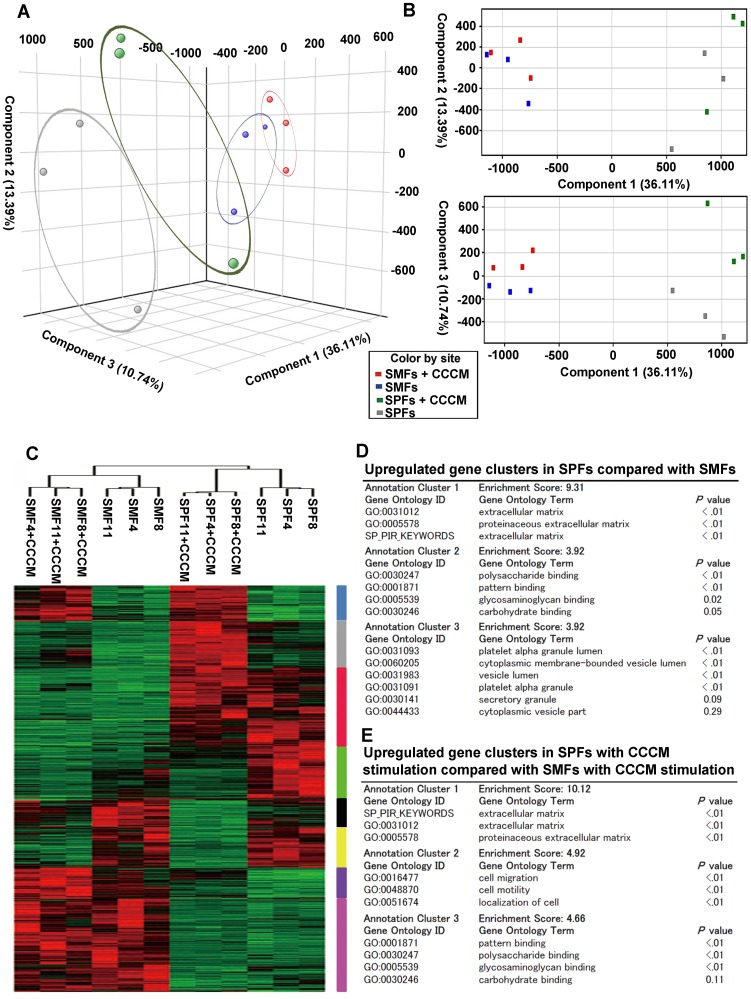
Gene expression profiles in subperitoneal fibroblasts (SPFs) and submucosal fibroblasts (SMFs) with and without cancer-cell-conditioned medium (CCCM) stimulation. (A) Red is the microarray profile in SMFs with CCCM stimulation, blue is SMFs without CCCM stimulation, green is SPFs with CCCM stimulation, and silver is SPFs without CCCM stimulation. Three-dimensional representation of principal component analysis (PCA) component 1, 2, and 3. (B) Two dimensional representation of PCA components 1 and 2 (upper), and PCA components 1 and 3 (lower). Fibroblasts formed independent clusters, depending on histoanatomical site and the presence of CCCM stimulation. (C) Supervised cluster analysis in fibroblasts also revealed distinct clusters depending on histoanatomical site and the presence of CCCM stimulation. (D) Gene ontology analysis of upregulated genes in SPFs compared with SMFs. (E) Gene ontology analysis of genes upregulated in SPFs with CCCM stimulation, compared with SMFs with CCCM stimulation. Most of the genes with increased expressions in SPFs were retained after CCCM stimulation; however, there were some differences, and the order of annotation clusters were changed after CCCM stimulation.

To assess the phenotypical differences between SPFs and SMFs, the gene expression profiles of fibroblasts with or without CCCM stimulation were compared. PCA revealed 4 distinct clusters, depending on their origin and CCCM stimulation, which overcame the individual variation (Figure 3A and B). Supervised cluster analysis also revealed 4 distinct clusters (Figure 3C). This indicated the phenotypical difference in fibroblasts within the colonic wall. And this difference depended on the histoanatomical location. Furthermore, the reaction to CCCM stimulation was also different.

Next, we compared gene expression profiles in these fibroblasts with and without CCCM stimulation, separately (Figure 3D–E). Data from SPFs without CCCM stimulation were enriched by the gene ontology (GO) terms “extracellular matrix” and “proteinaceous extracellular matrix”, which formed annotation cluster 1. Major expracellular matrix (ECM) components of collagens (COL1A1, COL4A1, COL4A2, COL5A1, and COL16A1), laminin, or fibronectin were included in this cluster. Moreover, gene expression related to components that bind to the ECM was also upregulated in SPFs and formed annotation cluster 2. Annotation cluster 3 was enriched for GO terms associated with “granules” or “vesicles” (Figure 3D and Table S3). This result suggested the gene expression profile associated with basic function in fibroblasts is different between SPFs and SMFs within the colonic wall. The top 20 genes highly expressed in SPFs also included several ECM components. Genes associated with fibrogenesis or the myofibroblastic differentiation of FLI1 and NOX4 were also found in the top 20 genes (Table S4). Among other highly expressed genes in SPFs without CCCM stimulation, we found POSN, SPARC, or COL4A1, which are known to be highly expressed in the cancer stroma; and many of these are prognostic factors [11],[16],[17].

Most of the genes with increased expressions in SPFs without CCCM stimulation were also retained in the presence of CCCM stimulation; however, there were some differences (Figure 3D–E, Table S5–6). In GO analysis of SPFs with CCCM stimulation, the order of annotation clusters were changed compared to that seen in SPFs without CCCM stimulation. Among the top 20 genes, 13 genes were conserved and 7 genes were replaced. These results suggest a difference in the reaction to CCCM stimulation between SPFs and SMFs. The existence of SPFs-specific genes that are upregulated after CCCM stimulation was estimated.

**Figure 4:**
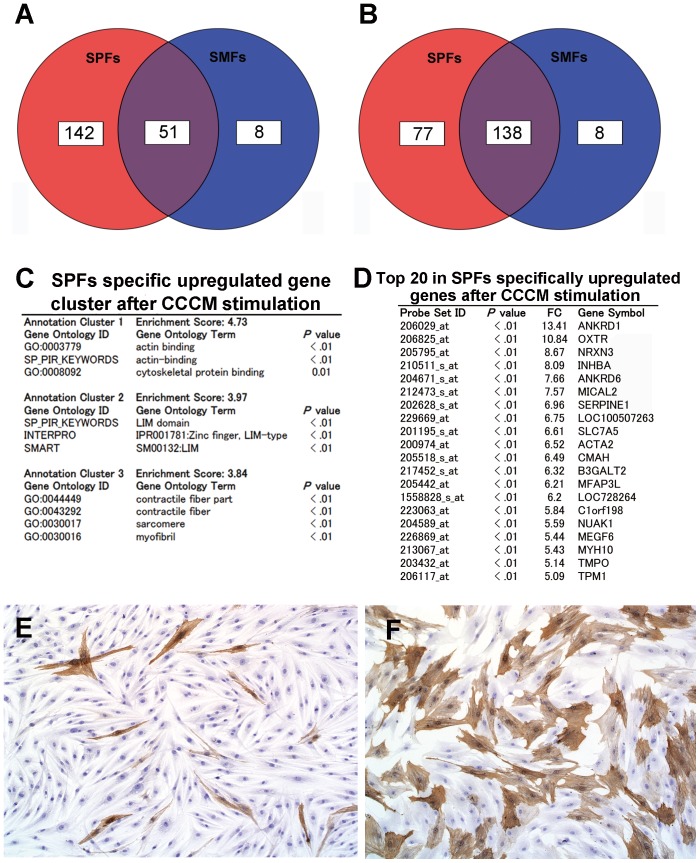
Gene modification in subperitoneal fibroblasts (SPFs) after cancer-cell-conditioned medium (CCCM) stimulation. (A) Genes upregulated by CCCM stimulation. (B) Genes downregulated by CCCM stimulation. (C) Top 3 annotation clusters in gene ontology analysis of SPFs-specific genes upregulated by CCCM stimulation. (D) Top 20 genes upregulated specifically in SPFs after CCCM stimulation. (E) Immunocytochemical α-SMA expression in SMFs after CCCM stimulation. (F) Immunocytochemical α-SMA expression in SPFs after CCCM stimulation. α-SMA expression was upregulated specifically in SPFs after CCCM stimulation (see also Figure S2).

We then analyzed these genes to establish the biological characteristics of SPFs after exposure to CCCM. A Venn diagram revealed 193 upregulated genes in SPFs and 59 in SMFs after CCCM stimulation. Of these, 51 were commonly upregulated both in SPFs and SMFs, 142 were SPFs specific, and only 8 were SMFs specific (Figure 4A). We then also focused on downregulated genes, and discovered 215 in SPFs and 146 in SMFs. Of these, 138 were commonly downregulated both in SPFs and SMFs, 77 were SPFs specific, and only 8 were SMFs specific (Figure 4B). These results suggested that SPFs showed more variable alteration in gene expression after CCCM stimulation. GO term analysis of SPF-specific genes downregulated after CCCM stimulation did not revealed any annotation cluster over 3.0 of the enrichment score (data not shown). In contrast, GO term analysis of SPFs-specific genes upregulated after CCCM stimulation revealed that terms such as “actin binding”, “cytoskeletal binding protein”, “contractile fiber”, “LIM domain”, “contractile fiber part”, “sarcomere”, and “myofibril” formed annotation cluster 1–3 (Figure 4C). Most of these genes were known to be related to cell contraction. Among the top 20 genes were many cytoskeletal or contractility associated genes. Surprisingly, ACTA2 that encodes α-SMA was upregulated specifically in SPFs after CCCM stimulation (Figure 4D). This result was confirmed by immunocytochemistry (Figure 4E–F). Morphometric analysis in immunocytochemical expression revealed that α-SMA expression was upregulated specifically and significantly in SPFs after CCCM stimulation in protein level (Figure S2, *P*<0.05). Variable upregulation and downregulation of genes after CCCM stimulation was a significant feature in SPFs. ACTA2 encoding α-SMA was included in this SPFs-specific upregulated gene set. Cancer associated fibroblasts (CAFs) include α-SMA-positive activated myofibroblasts. Together with the result of area-specific tissue microarrays, marked α-SMA expression in CMPI is depended on the sensitive character of SPFs which may associated with the difference in cancer microenvironment.

### SPFs Enhance Tumor Growth, Metastasis and Tumor Formation Ability more Strongly than do SMFs

**Figure 5:**
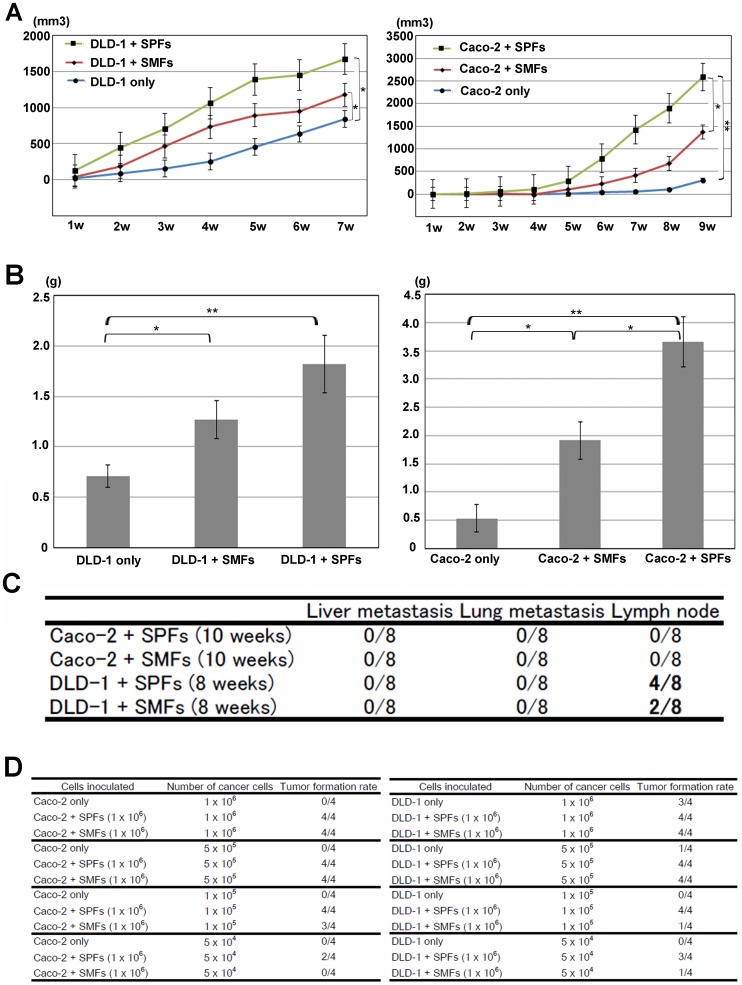
Subperitoneal fibroblasts (SPFs) actively contribute to cancer progression. (A, left) Xenograft tumor growth in mice injected with DLD-1 human colorectal cancer cells alone (blue line, 840.7±112.6 mm^3^ in 7 weeks), co-injected with DLD-1 cells and submucosal fibroblasts (SMFs; red line, 1178.0±177.6 mm^3^ in 7 weeks), and co-injected with DLD-1 cells and subperitoneal fibroblasts (SPFs; green line, 1672.8±214.7 mm^3^ in 7 weeks). The differences of tumor volume between DLD-1 cells alone and DLD-1 cells with SPFs, and between DLD-1 cells alone and DLD-1 cells with SMFs were statistically significant (*P*<0.05). (A, right) Xenograft tumor growth in mice injected with Caco-2 human colorectal cancer cells alone (blue line, 308.6±127.7 mm^3^ in 9 weeks), co-injected with Caco-2 cells and SMFs (red line, 1363.1±284.3 mm^3^ in 9 weeks), and co-injected with Caco-2 and SPFs (green line, 2595.1±349.5 mm^3^ in 9 weeks). The differences of tumor volume between Caco-2 cells alone and Caco-2 cells with SPFs (*P*<0.01), and between Caco-2 cells with SMFs and Caco-2 cells with SPFs (*P*<0.05) were statistically significant. Xenograft tumors derived from co-injection of cancer cells and SPFs grew faster than those derived from injection of cancer cells alone, or co-injection of cancer cells and SMFs. (B, left) Xenograft tumor weight in mice injected with DLD-1 cells alone was 0.71±0.11 g, co-injected with DLD-1 cells and SMFs was 1.27±0.19 g, and co-injected with DLD-1 cells and SPFs was 1.82±0.28 g in 8 weeks. The differences of tumor weight between DLD-1 cells alone and DLD-1 cells with SPFs (*P*<0.01), and between DLD-1 cells alone and DLD-1 cells with SMFs (*P*<0.05) were statistically significant. (B, right) Xenograft tumor weight in mice injected with Caco-2 cells alone was 0.53±0.24 g, co-injected with Caco-2 cells and SMFs was 1.91±0.34 g, and co-injected with Caco-2 cells and SPFs was 3.66±0.45 g in 10 weeks. The differences of tumor weight between DLD-1 cells alone and DLD-1 cells with SPFs (*P*<0.01), between DLD-1 cells alone and DLD-1 cells with SMFs (*P*<0.05), and between DLD-1 cells with SPFs and DLD-1 cells with SMFs (*P*<0.05) were statistically significant. Weights of xenograft tumors derived from co-injection of cancer cells with SPFs were higher than those of tumors derived from injection of cancer cells alone, or co-injection of cancer cells and SMFs (left: DLD-1, right: Caco-2). (C) Although the value did not reach statistical significance, xenograft tumors derived from co-injection of DLD-1 cells and SPFs showed twice the frequency of lymph node metastasis (n = 4) compared to those deriving from co-injection of DLD-1 cells and SMFs (n = 2). (D) Co-injection of cancer cells and SPFs result in enhanced tumor formation capacity. Results are presented as the mean ± SE of 8 mice (*P<0.05. **P<0.01).

To elucidate the functional differences of fibroblasts in the colonic wall, we injected human colorectal cancer cell lines Caco-2 or DLD-1 s.c., alone, or along with either SPFs or SMFs, into SCID mice. At 7 weeks after the injection, all mice demonstrated tumor formation. The growth of tumors arising from cancer cells injected along with SPFs grew faster than that arising from the injection of cancer cells alone or co-injection with SMFs (Figure 5A). The final weights of tumors arising from cancer cells co-injected with SPFs were also larger than those arising from the injection of cancer cells alone or from co-injection with SMFs (Figure 5B). Although the difference was not statistically significant, tumors arising from the co-injection of DLD-1 cells with SPFs more frequently resulted in lymph node metastasis than did those formed from the co-injection of DLD-1 cells with SMFs (Figure 5C).

Next, the cancer cells being injected were serially diluted and tumor formation was evaluated at 4 weeks after the injection, as described previously [13]. Comparison with mice injected with cancer cells alone or co-injected with cancer cells and SMFs, enhanced tumor formation was found in mice co-injected with cancer cells and SPFs, and tumor formation was observed even when the injected cells were diluted to 5×10^4^ (Caco-2) or 1×10^4^ (DLD-1). These results suggested that SPFs enhanced tumor growth, metastasis, and tumor formation capacity, in comparison with the effect of SMFs; these findings may be related to the peritoneal invasion dependent clinical outcome in colon cancer.

## Discussion

Fibroblasts are one of the most common types of stromal cells in connective tissue. Fibroblasts and loose connective tissue, which is one morphological type of connective tissue, are present throughout the body and contribute to the maintenance of the structural framework of most tissues, including the gastrointestinal tract [18]. Histologically, the gastrointestinal tract is composed of 5 layers that consist of the lamina propria, submucosa, muscular layer, subserosa, and serosa. Loose connective tissue and fibroblasts exist in every layer and have distinct physiological and pathological functions [19],[20].

SPFs are known to produce peritoneal fluid and facilitate appropriate functioning of intra-abdominal organs [21]. Previous reports have shown that the marked contractile ability in SPFs was implicated in the colonic strictures in Crohn’s disease [22],[23]. In the field of peritoneal dialysis, SPFs have been reported to produce growth factors, cytokines, or chemokines in response to TGF-β stimulation and have been implicated in the failure of peritoneal dialysis [24]. However, the implication of SPFs in tumor progression is not known, and our study is the first to report the contribution of SPFs in tumor progression and metastasis that is dependent on peritoneal invasion in colon cancer.

Our findings seem to support the concept of microenvironmental regulation of cancer. The tumor microenvironment consists of distinct cell types, including fibroblasts, blood cells, vascular-originated cells, and more. They synergistically create a distinct microenvironment according to tumor progression, such as the core primary tumor microenvironment, the invasive tumor microenvironment, or the metastatic tumor microenvironment [9]. Area-specific tissue microarrays were very useful to expand this concept into the pathological phenomenon and biological study was then performed based on these results. Interestingly, the fibroblasts we obtained from the submucosal and subperitoneal tissues showed biological differences dependent on the histoanatomical site. In addition, our finding of marked phenotypical modification in SPFs suggests that fibroblasts from different histoanatomical sites show different reactions to cancer stimuli. We used Caco-2 with low tumorigenic and metastatic potential and DLD-1 with a higher tumorigenic and metastatic potential in this study. DLD-1 has been known to preferentially cause lymph node metastasis rather than lung or liver metastasis, and our data is in accordance with previous reports [25]–[28]. Together with the xenograft tumor results in which SPFs enhanced tumor growth and tumor formation in Caco-2 and DLD-1, and promote lymph node metastasis in DLD-1, we have clarified that this fibroblastic difference is implicated in colon cancer progression that is dependent on peritoneal invasion.

In general, fibroblasts within the tumor stroma, so-called CAFs, acquired a modified phenotype. CAFs are enriched in α-SMA positive active myofibroblasts and are known to play an active role in tumor progression [29],[30]. Residual fibroblasts are one of the sources of CAFs, and residual fibroblasts exposed to cancer stimulation show phenotypical modification. Although the tumor-promoting ability of CAFs has been reported to be diverse and dependent on cancer origin, intra-tumoral diversity has not been clear [31]. Our data suggests the physiological diversity of fibroblasts within one organ produces the intra-tumoral diversity of CAFs. Therefore, gene profiles in fibroblasts with and without cancer CCCM stimulation may provide new insights into their diversity in colon cancer.

We are speculating that a fibroblast subpopulation with tumor-promoting capacity can be enriched in the subperitoneal layer. Their original phenotype may include a previously reported CAFs marker, and variable gene modification in response to cancer stimuli could be a characteristic feature of tumor-promoting fibroblasts. Recently, activated proteins in CAFs have been considered to be a target of therapy [32]. However, not all kinds of CAFs may promote tumor progression [31]. Our gene expression profile data in SPFs with and without CCCM stimulation may also be useful for future stromal-target therapy. SPFs with robust tumor promotion ability showed higher gene expression associated with an ECM component, and marked gene upregulation associated with cell contraction, including α-SMA, after CCCM stimulation. Recently both stromal-cell contractile ability and ECM stiffness have been reported to influence epithelial cell migration and invasion. Also, α-SMA is one of the representative markers of CAFs and myofibroblasts, and its expression is associated with biological contractile ability. Furthermore, α-SMA expression in tumor stroma was reported to be a prognostic factor in colorectal cancer. Therefore, our result suggests the importance of mechanotransduction theory in the study of the tumor microenvironment [33]–[35].

From the first categorization efforts reported by Lockhart-Mummery, primary colon cancer has been consistently stratified based on the extent of its spread into the bowel wall [36]. More recent pathological investigations have revealed that peritoneal invasion is a prognostic factor, and is a candidate for discriminating high-risk stage II colon cancer, and those patients who may receive benefit from post-operative therapy [3],[37],[38]. We and others reported the utility of elastica stain for the objective diagnosis of peritoneal invasion. We have proven that objective identification of peritoneal invasion is also useful for investigating biological phenomena specifically occurring in this tumor microenvironment [39]. Recently, Liang et al. proposed that pT3 tumors with ELI should be subdivided into further categories like pT3b [6]. Our findings support the subdivision of cases with ELI from those without ELI, and the diversity of the fibroblasts could be one factor associated with frequent metastases in cases with ELI.

In conclusion, fibrosis with α-SMA expression is a significant feature of the cancer microenvironment formed by peritoneal invasion in human colon cancer. The biological features and functions of fibroblasts in the subperitoneal tissue are different from those in submucosal tissue, and their phenotypical modification by cancer stimuli and contribution to tumor growth and metastasis are also different. Specifically, SPFs from the subperitoneal tissue showed characteristic biological features of a marked ECM component and contractile-associated gene expression, and functions that accelerate tumor formation and metastasis. Considering these comprehensive pathological and biological data, we propose that CMPI is a special microenvironment that promotes tumor growth and metastasis. In CMPI, SPFs and cancer cells interaction play an active and crucial role in tumor progression.

## Supporting Information

Figure S1 Biological characteristics of subperitoneal fibroblasts and submucosal fibroblasts. (A) Immunocytochemical staining for vimentin in SPFs. (B) Immunocytochemical staining for vimentin in SMFs. (C) Flow cytometric analysis of SPFs and SMFs. (D) Immunocytochemical staining of SPFs and SMFs. Protein expression results were positive for vimentin and CD105, and negative for an epithelial marker (AE1/3), a neural marker (S-100), mesothelial markers (calretinin, CK8), endothelial markers (CD31, 34), and lymphocyte and monocyte markers (CD3, 14, 20, 45, 68), suggesting that the obtained cells were fibroblasts. We found weak α-SMA expression in a few SPFs and SMFs. (E) Growth curve of SPFs (blue) and SMFs (red). SPFs showed significantly longer doubling time than did SMFs (*P*<0.01)**.** Results are presented as the mean ± SE of triplicate measurements (**P<0.01).
(TIF)

Figure S2 Morphometric analysis of immunocytochemical α-SMA expression. α-SMA expression was upregulated specifically and significantly in SPFs after cancer-cell-conditioned medium (CCCM) stimulation. Results are presented as the mean ± SE of 3 experiments (*P<0.05).
(TIF)

Table S1 Patient Characteristics Entered into Area-Specific Tissue Microarray.
(DOCX)

Table S2 Primary antibodies used in this study.
(DOCX)

Table S3 Upregulated gene clusters and composing genes in SPFs compared with SMFs.
(DOCX)

Table S4 Top 20 genes in SPFs compared with SMFs.
(DOCX)

Table S5 Upregulated gene clusters in SPFs with cancer-cell-conditioned medium (CCCM) stimulation compared with SMFs with CCCM stimulation.
(DOCX)

Table S6 Top 20 upregulated genes in SPFs with cancer cell-conditioned medium (CCCM) stimulation compared with SMFs with CCCM stimulation.
(DOCX)

Materials and Methods S1 Supplementary Materials and Methods.
(DOCX)
