# Supplementary figures and images for: Human Subperitoneal Fibroblast and Cancer Cell Interaction Creates Microenvironment That Enhances Tumor Progression and Metastasis
Source: PLoS One. 2014 Feb 4;9(2):e88018. doi: 10.1371/journal.pone.0088018 (PMC3913740; doi:10.1371/journal.pone.0088018)

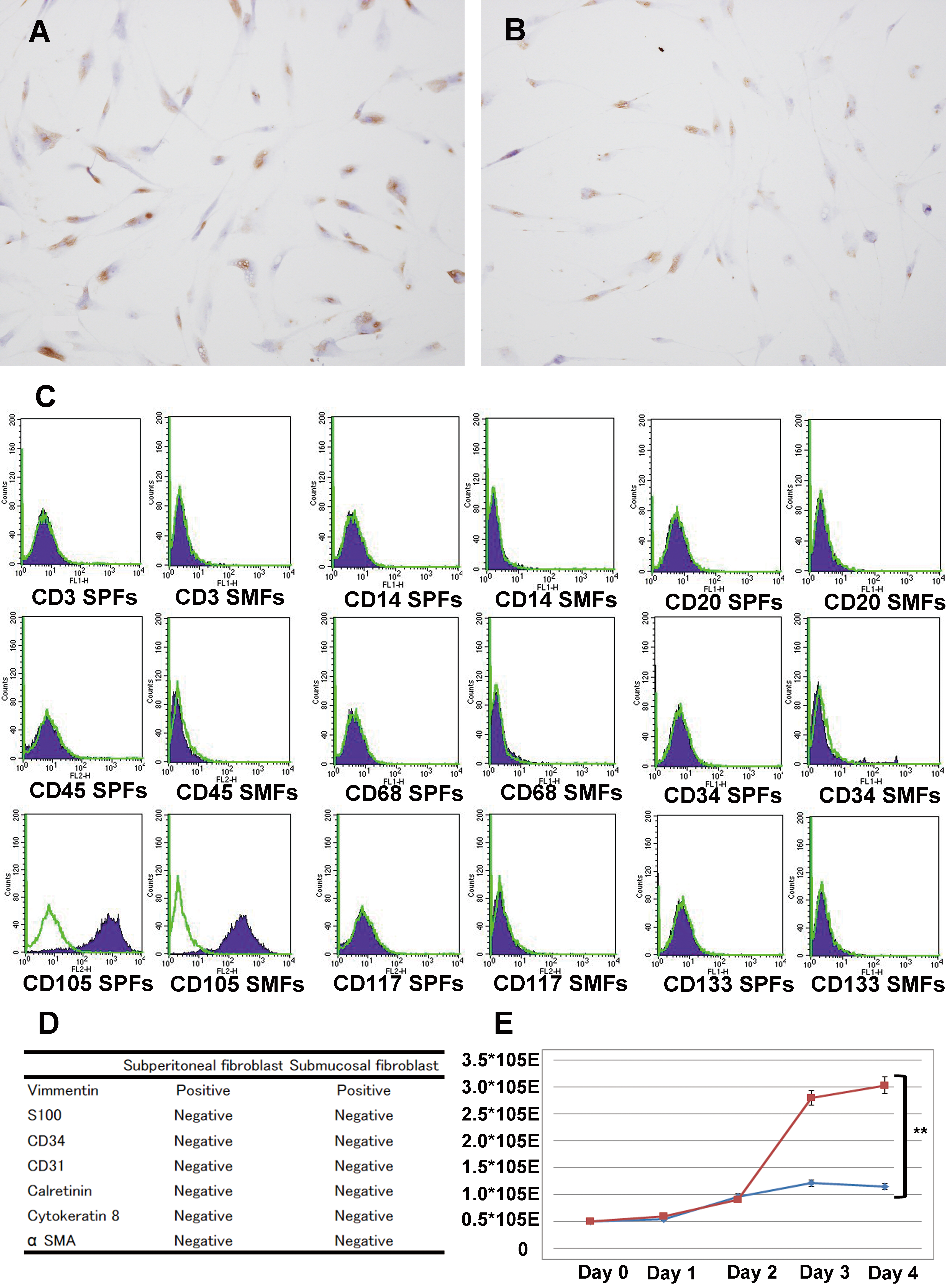

Supplement: Figure S1 — Biological characteristics of subperitoneal fibroblasts and submucosal fibroblasts. (A) Immunocytochemical staining for vimentin in SPFs. (B) Immunocytochemical staining for vimentin in SMFs. (C) Flow cytometric analysis of SPFs and SMFs. (D) Immunocytochemical staining of SPFs and SMFs. Protein expression results were positive for vimentin and CD105, and negative for an epithelial marker (AE1/3), a neural marker (S-100), mesothelial markers (calretinin, CK8), endothelial markers (CD31, 34), and lymphocyte and monocyte markers (CD3, 14, 20, 45, 68), suggesting that the obtained cells were fibroblasts. We found weak α-SMA expression in a few SPFs and SMFs. (E) Growth curve of SPFs (blue) and SMFs (red). SPFs showed significantly longer doubling time than did SMFs (P<0.01). Results are presented as the mean ± SE of triplicate measurements (**P<0.01). (TIF) [file pone.0088018.s001.tif]

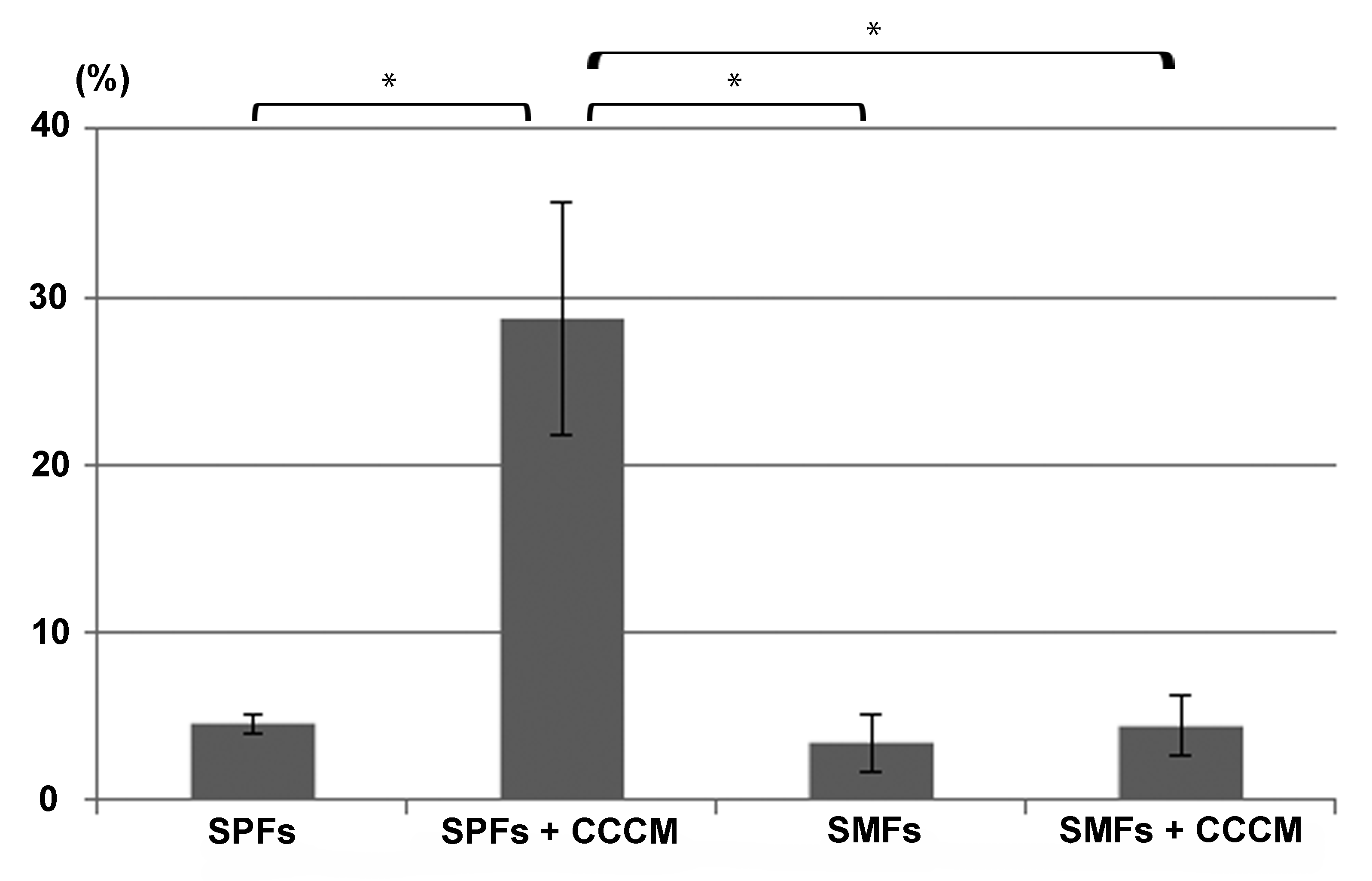

Supplement: Figure S2 — Morphometric analysis of immunocytochemical α-SMA expression. α-SMA expression was upregulated specifically and significantly in SPFs after cancer-cell-conditioned medium (CCCM) stimulation. Results are presented as the mean ± SE of 3 experiments (*P<0.05). (TIF) [file pone.0088018.s002.tif]
