# Supplementary material for: Human Subperitoneal Fibroblast and Cancer Cell Interaction Creates Microenvironment That Enhances Tumor Progression and Metastasis
Source: PLoS One. 2014 Feb 4;9(2):e88018. doi: 10.1371/journal.pone.0088018 (PMC3913740; doi:10.1371/journal.pone.0088018)
Supplement: Table S1 — Patient Characteristics Entered into Area-Specific Tissue Microarray. (DOCX) [file pone.0088018.s003.docx]

| **Table S1. *Patient Characteristics Entered into Area-Specific Tissue Microarray*** | | |
| --- | --- | --- |
| Age | Years (mean±SD) | 63.6±10.3 |
| Gender | Male | 88 |
|  | Female | 61 |
| Tumor location | Right | 67 |
|  | Left | 82 |
| TNM Stage | Stage II | 49 |
|  | Stage III | 53 |
|  | Stage IV | 47 |
| Curative potential of resection | Curative | 110 |
|  | Palliative | 39 |
| Lymph node metastasis | Positive | 57 |
|  | Negative | 92 |
| Histoloigcal type | Well or Moderate | 126 |
|  | Others | 23 |
| pT stage | pT3 | 91 |
|  | pT4a | 58 |
