# Supplementary material for: Human Subperitoneal Fibroblast and Cancer Cell Interaction Creates Microenvironment That Enhances Tumor Progression and Metastasis
Source: PLoS One. 2014 Feb 4;9(2):e88018. doi: 10.1371/journal.pone.0088018 (PMC3913740; doi:10.1371/journal.pone.0088018)
Supplement: Table S2 — Primary antibodies used in this study. (DOCX) [file pone.0088018.s004.docx]

| **Table S2. Primary antibodies used in this study** | | | |
| --- | --- | --- | --- |
| Antibody | Clone | Company | Use |
| CD3 | UCHT1 | DakoCytomation, Glostrup, Denmark | IHC, FC |
| CD31 | JC70A | DakoCytomation, Glostrup, Denmark | IHC |
| CD68 | PG-M1 | DakoCytomation, Glostrup, Denmark | IHC |
| α-SMA | 1A4 | Sigma, Saint Louis, USA | IHC |
| Vimentin | Vim 3B4 | DakoCytomation, Glostrup, Denmark | IHC |
| S100 | polyclonal | DakoCytomation, Glostrup, Denmark | IHC |
| CD14, FITC-Conjugated | 61D3 | eBioscience, CA, USA | FC |
| CD20, FITC-Conjugated | 2H7 | eBioscience, CA, USA | FC |
| CD34 | QBEnd 10 | DakoCytomation, Glostrup, Denmark | FC |
| CD68, FITC-Conjugated | KP1 | DakoCytomation, Glostrup, Denmark | FC |
| CD133 | AC133 | Miltenyi Biotec, Glabach, Germany | FC |
| CD105 | SN6h | DakoCytomation, Glostrup, Denmark | FC |
| CD117, FITC-Conjugated | 104D2 | DakoCytomation, Glostrup, Denmark | FC |
| CD45, FITC-Conjugated | HI30 | eBioscience, CA, USA | FC |
| Abbreviations: IHC, immunohistochemicstry; FC, flow cytometry. | | | |
