# Supplementary material for: Human Subperitoneal Fibroblast and Cancer Cell Interaction Creates Microenvironment That Enhances Tumor Progression and Metastasis
Source: PLoS One. 2014 Feb 4;9(2):e88018. doi: 10.1371/journal.pone.0088018 (PMC3913740; doi:10.1371/journal.pone.0088018)
Supplement: Table S3 — Upregulated gene clusters and composing genes in SPFs compared with SMFs. (DOCX) [file pone.0088018.s005.docx]

| **Table S3. Upregulated gene clusters and composing genes in SPFs compared with SMFs** | | | | | |
| --- | --- | --- | --- | --- | --- |
| **Upregulated gene clusters in SPFs** | | |  | **Genes composing Annotation Clusters** | |
| **Annotation Cluster 1** | **Enrichment Score: 9.31** |  |  | **Annotation Cluster 1** |  |
| **Gene Ontology ID** | **Gene Ontology Term** | ***P* value** |  | **Probe Set ID** | **Gene Name** |
| GO:0031012 | extracellular matrix | < .01 |  | 226997_at | ADAM metallopeptidase with thrombospondin |
| GO:0005578 | proteinaceous extracellular matrix | < .01 |  |  | type 1 motif, 12 |
| SP_PIR_KEYWORDS | extracellular matrix | < .01 |  | 219087_at, 224396_s_at | asporin |
|  |  |  |  | 201261_x_at, 213905_x_at | biglycan |
| **Annotation Cluster 2** | **Enrichment Score: 3.92** |  |  | 205713_s_at | cartilage oligomeric matrix protein |
| **Gene Ontology ID** | **Gene Ontology Term** | ***P* value** |  | 209395_at, 209396_s_at | chitinase 3-like 1 (cartilage glycoprotein-39) |
| GO:0030247 | polysaccharide binding | < .01 |  | 202310_s_at | collagen, type I, alpha 1 |
| GO:0001871 | pattern binding | < .01 |  | 211981_at, 211980_at | collagen, type IV, alpha 1 |
| GO:0005539 | glycosaminoglycan binding | 0.02 |  | 211964_at | collagen, type IV, alpha 2 |
| GO:0030246 | carbohydrate binding | 0.05 |  | 203325_s_at, 212489_at, 212488_at | collagen, type V, alpha 1 |
|  |  |  |  | 204345_at | collagen, type XVI, alpha 1 |
| **Annotation Cluster 3** | **Enrichment Score: 3.92** |  |  | 206101_at | extracellular matrix protein 2, female organ |
| **Gene Ontology ID** | **Gene Ontology Term** | ***P* value** |  |  | and adipocyte specific |
| GO:0031093 | platelet alpha granule lumen | < .01 |  | 214701_s_at, 214702_at | fibronectin 1 |
| GO:0060205 | cytoplasmic membrane-bounded vesicle lumen | < .01 |  | 227048_at | laminin, alpha 1 |
| GO:0031983 | vesicle lumen | < .01 |  | 223690_at | latent transforming growth factor beta |
| GO:0031091 | platelet alpha granule | < .01 |  |  | binding protein 2 |
| GO:0030141 | secretory granule | 0.09 |  | 202291_s_at | matrix Gla protein |
| GO:0044433 | cytoplasmic vesicle part | 0.29 |  | 213764_s_at, 209758_s_at, 213765_at | microfibrillar associated protein 5 |
|  |  |  |  | 203417_at | microfibrillar-associated protein 2 |
| **Annotation Cluster 4** | **Enrichment Score: 3.32** |  |  | 1555778_a_at, 210809_s_at | periostin, osteoblast specific factor |
| **Gene Ontology ID** | **Gene Ontology Term** | ***P* value** |  | 212667_at | secreted protein, acidic, cysteine-rich |
| GO:0045935 | positive regulation of nucleobase, nucleoside, | 0.10 |  |  | (osteonectin) |
|  | nucleotide and nucleic acid metabolic process |  |  | 202363_at | sparc/osteonectin, cwcv and kazal-like |
| GO:0045893 | positive regulation of transcription, | 0.07 |  |  | domains proteoglycan (testican) 1 |
|  | DNA-dependent |  |  | 235086_at, 201108_s_at, 201107_s_at | thrombospondin 1 |
| GO:0031328 | positive regulation of cellular biosynthetic | 0.06 |  | 209747_at | transforming growth factor, beta 3 |
|  | process |  |  | 204619_s_at, 215646_s_at | versican |
| GO:0051254 | positive regulation of RNA metabolic process | 0.05 |  | **Annotation Cluster 2** |  |
| GO:0009891 | positive regulation of biosynthetic process | 0.06 |  | **Probe Set ID** | **Gene Name** |
| GO:0051173 | positive regulation of nitrogen compound | 0.05 |  | 201261_x_at, 213905_x_at | biglycan |
|  | metabolic process |  |  | 205713_s_at | cartilage oligomeric matrix protein |
| GO:0010557 | positive regulation of macromolecule | 0.05 |  | 203325_s_at, 212489_at, 212488_at | collagen, type V, alpha 1 |
|  | biosynthetic process |  |  | 206101_at | extracellular matrix protein 2, female organ |
| GO:0045941 | positive regulation of transcription | 0.06 |  |  | and adipocyte specific |
| GO:0045944 | positive regulation of transcription from | 0.05 |  | 214701_s_at, 214702_at | fibronectin 1 |
|  | RNA polymerase II promoter |  |  | 203821_at | heparin-binding EGF-like growth factor |
| GO:0010628 | positive regulation of gene expression | 0.06 |  | 1555778_a_at, 210809_s_at | periostin, osteoblast specific factor |
| GO:0010604 | positive regulation of macromolecule | 0.09 |  | 206007_at | proteoglycan 4 |
|  | metabolic process |  |  | 227480_at | sushi domain containing 2 |
|  |  |  |  | 235086_at, 201108_s_at, 201107_s_at | thrombospondin 1 |
| **Annotation Cluster 5** | **Enrichment Score: 2.95** |  |  | 204619_s_at, 215646_s_at | versican |
| **Gene Ontology ID** | **Gene Ontology Term** | ***P* value** |  | 209395_at, 209396_s_at | chitinase 3-like 1 (cartilage glycoprotein-39) |
| GO:0016477 | cell migration | 0.05 |  | 205100_at | glutamine-fructose-6-phosphate |
| GO:0048870 | cell motility | 0.08 |  |  | transaminase 2 |
| GO:0051674 | localization of cell | 0.08 |  | **Annotation Cluster 3** |  |
|  |  |  |  | **Probe Set ID** | **Gene Name** |
| **Annotation Cluster 6** | **Enrichment Score: 2.77** |  |  | 214701_s_at, 214702_at | fibronectin 1 |
| **Gene Ontology ID** | **Gene Ontology Term** | ***P* value** |  | 212667_at | secreted protein, acidic, cysteine-rich |
| GO:0001568 | blood vessel development | 0.06 |  |  | (osteonectin) |
| GO:0001944 | vasculature development | 0.07 |  | 201859_at, 201858_s_at | serglycin |
| GO:0048514 | blood vessel morphogenesis | 0.16 |  | 200986_at | serpin peptidase inhibitor, clade G |
|  |  |  |  |  | (C1 inhibitor), member 1 |
| **Annotation Cluster 7** | **Enrichment Score: 2.76** |  |  | 235086_at, 201108_s_at, 201107_s_at | thrombospondin 1 |
| **Gene Ontology ID** | **Gene Ontology Term** | ***P* value** |  | 209561_at | thrombospondin 3 |
| UP_SEQ_FEATURE | domain:TSP C-terminal | 0.30 |  | 209747_at | transforming growth factor, beta 3 |
| UP_SEQ_FEATURE | repeat:TSP type-3 4 | 0.30 |  | 209238_at | syntaxin 3 |
| UP_SEQ_FEATURE | repeat:TSP type-3 3 | 0.30 |  |  |  |
| UP_SEQ_FEATURE | repeat:TSP type-3 2 | 0.30 |  |  |  |
| UP_SEQ_FEATURE | repeat:TSP type-3 1 | 0.30 |  |  |  |
| UP_SEQ_FEATURE | repeat:TSP type-3 8 | 0.30 |  |  |  |
| UP_SEQ_FEATURE | repeat:TSP type-3 7 | 0.30 |  |  |  |
| UP_SEQ_FEATURE | repeat:TSP type-3 6 | 0.30 |  |  |  |
| UP_SEQ_FEATURE | repeat:TSP type-3 5 | 0.30 |  |  |  |
| INTERPRO | IPR008859:Thrombospondin, C-terminal | 0.53 |  |  |  |
| INTERPRO | IPR003367:Thrombospondin, type 3-like repeat | 0.53 |  |  |  |
| INTERPRO | IPR017897:Thrombospondin, type 3 repeat | 0.53 |  |  |  |
| UP_SEQ_FEATURE | domain:EGF-like 2; calcium-binding | 0.89 |  |  |  |
|  |  |  |  |  |  |
| **Annotation Cluster 8** | **Enrichment Score: 2.71** |  |  |  |  |
| **Gene Ontology ID** | **Gene Ontology Term** | ***P* value** |  |  |  |
| SP_PIR_KEYWORDS | hydroxylysine | 0.02 |  |  |  |
| SP_PIR_KEYWORDS | triple helix | 0.02 |  |  |  |
| GOTERM_CC_FAT | GO:0005581~collagen | 0.02 |  |  |  |
| SP_PIR_KEYWORDS | hydroxyproline | 0.03 |  |  |  |
| SP_PIR_KEYWORDS | hydroxylation | 0.04 |  |  |  |
| UP_SEQ_FEATURE | region of interest:Triple-helical region | 0.34 |  |  |  |
| INTERPRO | IPR008160:Collagen triple helix repeat | 0.65 |  |  |  |
| SP_PIR_KEYWORDS | collagen | 0.29 |  |  |  |
|  |  |  |  |  |  |
| **Annotation Cluster 9** | **Enrichment Score: 2.52** |  |  |  |  |
| **Gene Ontology ID** | **Gene Ontology Term** | ***P* value** |  |  |  |
| UP_SEQ_FEATURE | domain:TSP N-terminal | 0.34 |  |  |  |
| INTERPRO | IPR003129:Laminin G, thrombospondin-type, | 0.46 |  |  |  |
|  | N-terminal |  |  |  |  |
| SMART | SM00210:TSPN | 0.45 |  |  |  |
|  |  |  |  |  |  |
| **Annotation Cluster 10** | **Enrichment Score: 2.15** |  |  |  |  |
| **Gene Ontology ID** | **Gene Ontology Term** | ***P* value** |  |  |  |
| GO:0030335 | positive regulation of cell migration | 0.16 |  |  |  |
| GO:0030334 | regulation of cell migration | 0.16 |  |  |  |
| GO:0040017 | positive regulation of locomotion | 0.18 |  |  |  |
| GO:0051272 | positive regulation of cell motion | 0.18 |  |  |  |
| GO:0040012 | regulation of locomotion | 0.22 |  |  |  |
| GO:0051270 | regulation of cell motion | 0.22 |  |  |  |
