# Supplementary material for: Human Subperitoneal Fibroblast and Cancer Cell Interaction Creates Microenvironment That Enhances Tumor Progression and Metastasis
Source: PLoS One. 2014 Feb 4;9(2):e88018. doi: 10.1371/journal.pone.0088018 (PMC3913740; doi:10.1371/journal.pone.0088018)
Supplement: Table S4 — Top 20 genes in SPFs compared with SMFs. (DOCX) [file pone.0088018.s006.docx]

| **Table S4. Top 20 genes in SPFs compared with SMFs** | | | | |
| --- | --- | --- | --- | --- |
| **Probe Set ID** | ***P* value** | **FC** | **Gene Symbol** | **Gene Title** |
| 203824_at | < .01 | 520.1 | TSPAN8 | tetraspanin 8 |
| 223121_s_at | < .01 | 253.4 | SFRP2 | secreted frizzled-related protein 2 |
| 200606_at | < .01 | 243.0 | DSP | desmoplakin |
| 206067_s_at | < .01 | 94.1 | WT1 | Wilms tumor 1 |
| 205475_at | < .01 | 78.9 | SCRG1 | stimulator of chondrogenesis 1 |
| 213764_s_at | < .01 | 69.3 | MFAP5 | microfibrillar associated protein 5 |
| 206858_s_at | < .01 | 60.8 | HOXC6 | homeobox C6 |
| 201596_x_at | < .01 | 49.1 | KRT18 | keratin 18 |
| 202291_s_at | < .01 | 48.6 | MGP | matrix Gla protein |
| 204236_at | < .01 | 47.3 | FLI1 | Friend leukemia virus integration 1 |
| 204337_at | < .01 | 45.5 | RGS4 | regulator of G-protein signaling 4 |
| 229674_at | < .01 | 40.1 | SERTAD4 | SERTA domain containing 4 |
| 219773_at | < .01 | 39.4 | NOX4 | NADPH oxidase 4 |
| 230660_at | < .01 | 33.4 | SERTAD4 | SERTA domain containing 4 |
| 226961_at | < .01 | 30.1 | PRR15 | proline rich 15 |
| 235085_at | < .01 | 29.2 | SGK223 | homolog of rat pragma of Rnd2 |
| 205713_s_at | < .01 | 28.3 | COMP | cartilage oligomeric matrix protein |
| 210942_s_at | < .01 | 24.3 | ST3GAL6 | ST3 beta-galactoside alpha-2,3-sialyltransferase 6 |
| 202286_s_at | < .01 | 20.9 | TACSTD2 | tumor-associated calcium signal transducer 2 |
| 209396_s_at | < .01 | 20.7 | CHI3L1 | chitinase 3-like 1 (cartilage glycoprotein-39) |
