# Supplementary material for: Human Subperitoneal Fibroblast and Cancer Cell Interaction Creates Microenvironment That Enhances Tumor Progression and Metastasis
Source: PLoS One. 2014 Feb 4;9(2):e88018. doi: 10.1371/journal.pone.0088018 (PMC3913740; doi:10.1371/journal.pone.0088018)
Supplement: Table S5 — Upregulated gene clusters in SPFs with cancer-cell-conditioned medium (CCCM) stimulation compared with SMFs with CCCM stimulation. (DOCX) [file pone.0088018.s007.docx]

| **Table S5. Upregulated gene clusters in SPFs with cancer-cell-conditioned medium (CCCM) stimulation compared with SMFs with CCCM stimulation** | | | | | |
| --- | --- | --- | --- | --- | --- |
| **Upregulated gene clusters in SPFs with CCCM stimulation** | | |  | **Genes composing Annotation Clusters** | |
| **Annotation Cluster 1** | **Enrichment Score: 10.12** |  |  | **Annotation Cluster 1** |  |
| **Gene Ontology ID** | **Gene Ontology Term** | ***P* value** |  | **Probe Set ID** | **Gene Name** |
| SP_PIR_KEYWORDS | extracellular matrix | < .01 |  | 226311_at | ADAM metallopeptidase with thrombospondin |
| GO:0031012 | extracellular matrix | < .01 |  |  | type 1 motif, 2 |
| GO:0005578 | proteinaceous extracellular matrix | < .01 |  | 219087_at | asporin |
|  |  |  |  | 213905_x_at, 201261_x_at | biglycan |
| **Annotation Cluster 2** | **Enrichment Score: 4.92** |  |  | 205713_s_at | cartilage oligomeric matrix protein |
| **Gene Ontology ID** | **Gene Ontology Term** | ***P* value** |  | 243864_at | coiled-coil domain containing 80 |
| GO:0016477 | cell migration | < .01 |  | 202311_s_at, 202310_s_at | collagen, type I, alpha 1 |
| GO:0048870 | cell motility | < .01 |  | 211981_at, 211980_at | collagen, type IV, alpha 1 |
| GO:0051674 | localization of cell | < .01 |  | 211966_at, 211964_at | collagen, type IV, alpha 2 |
|  |  |  |  | 212489_at, 203325_s_at, 212488_at | collagen, type V, alpha 1 |
| **Annotation Cluster 3** | **Enrichment Score: 4.66** |  |  | 221900_at | collagen, type VIII, alpha 2 |
| **Gene Ontology ID** | **Gene Ontology Term** | ***P* value** |  | 204345_at | collagen, type XVI, alpha 1 |
| GO:0001871 | pattern binding | < .01 |  | 209101_at | connective tissue growth factor |
| GO:0030247 | polysaccharide binding | < .01 |  | 206101_at | extracellular matrix protein 2, female organ |
| GO:0005539 | glycosaminoglycan binding | < .01 |  |  | and adipocyte specific |
| GO:0030246 | carbohydrate binding | 0.11 |  | 214702_at, 214701_s_at | fibronectin 1 |
|  |  |  |  | 227048_at | laminin, alpha 1 |
| **Annotation Cluster 4** | **Enrichment Score: 4.28** |  |  | 213764_s_at, 213765_at, 209758_s_at | microfibrillar associated protein 5 |
| **Gene Ontology ID** | **Gene Ontology Term** | ***P* value** |  | 203417_at | microfibrillar-associated protein 2 |
| SP_PIR_KEYWORDS | LIM domain | < .01 |  | 1555778_a_at, 210809_s_at | periostin, osteoblast specific factor |
| INTERPRO | IPR001781:Zinc finger, LIM-type | 0.02 |  | 212667_at | secreted protein, acidic, cysteine-rich |
| SMART | SM00132:LIM | 0.02 |  |  | (osteonectin) |
|  |  |  |  | 202363_at | sparc/osteonectin, cwcv and kazal-like |
| **Annotation Cluster 5** | **Enrichment Score: 3.64** |  |  |  | domains proteoglycan (testican) 1 |
| **Gene Ontology ID** | **Gene Ontology Term** | ***P* value** |  | 215646_s_at, 204619_s_at | versican |
| GO:0001568 | blood vessel development | 0.02 |  | 223690_at | latent transforming growth factor beta binding |
| GO:0001944 | vasculature development | 0.02 |  |  | protein 2 |
| GO:0048514 | blood vessel morphogenesis | 0.04 |  | 202291_s_at | matrix Gla protein |
|  |  |  |  | 201108_s_at, 201107_s_at, 235086_at | thrombospondin 1 |
| **Annotation Cluster 6** | **Enrichment Score: 3.59** |  |  | **Annotation Cluster 2** |  |
| **Gene Ontology ID** | **Gene Ontology Term** | ***P* value** |  | **Probe Set ID** | **Gene Name** |
| GO:0031093 | platelet alpha granule lumen | < .01 |  | 40148_at, 212985_at, 213419_at | amyloid beta (A4) precursor protein-binding, |
| GO:0060205 | cytoplasmic membrane-bounded vesicle lumen | < .01 |  |  | family B, member 2 |
| GO:0031983 | vesicle lumen | < .01 |  | 203440_at, 203441_s_at | cadherin 2, type 1, N-cadherin (neuronal) |
| GO:0031091 | platelet alpha granule | < .01 |  | 212489_at, 203325_s_at, 212488_at | collagen, type V, alpha 1 |
| GO:0030141 | secretory granule | 0.10 |  | 209101_at | connective tissue growth factor |
| GO:0044433 | cytoplasmic vesicle part | 0.28 |  | 214702_at, 214701_s_at | fibronectin 1 |
|  |  |  |  | 1553613_s_at | forkhead box C1 |
| **Annotation Cluster 7** | **Enrichment Score: 3.46** |  |  | 203821_at | heparin-binding EGF-like growth factor |
| **Gene Ontology ID** | **Gene Ontology Term** | ***P* value** |  | 222899_at | integrin, alpha 11 |
| GO:0040012 | regulation of locomotion | 0.03 |  | 227048_at | laminin, alpha 1 |
| GO:0051270 | regulation of cell motion | 0.03 |  | 212372_at, 213067_at | myosin, heavy chain 10, non-muscle |
| GO:0030334 | regulation of cell migration | 0.04 |  | 211926_s_at | myosin, heavy chain 9, non-muscle |
|  |  |  |  | 204035_at | secretogranin II (chromogranin C) |
| **Annotation Cluster 8** | **Enrichment Score: 3.29** |  |  | 203789_s_at | sema domain, immunoglobulin domain (Ig), |
| **Gene Ontology ID** | **Gene Ontology Term** | ***P* value** |  |  | short basic domain, secreted, (semaphorin) 3C |
| GO:0005912 | adherens junction | < .01 |  | 221748_s_at | tensin 1 |
| GO:0070161 | anchoring junction | < .01 |  | 201108_s_at, 201107_s_at, 235086_at | thrombospondin 1 |
| GO:003005 | cell junction | 0.28 |  | **Annotation Cluster 3** |  |
|  |  |  |  | **Probe Set ID** | **Gene Name** |
| **Annotation Cluster 9** | **Enrichment Score: 3.23** |  |  | 213905_x_at, 201261_x_at | biglycan |
| **Gene Ontology ID** | **Gene Ontology Term** | ***P* value** |  | 205713_s_at | cartilage oligomeric matrix protein |
| GO:0048738 | cardiac muscle tissue development | 0.01 |  | 243864_at | coiled-coil domain containing 80 |
| GO:0014706 | striated muscle tissue development | 0.07 |  | 212489_at, 203325_s_at, 212488_at | collagen, type V, alpha 1 |
| GO:0060537 | muscle tissue development | 0.07 |  | 209101_at | connective tissue growth factor |
|  |  |  |  | 206101_at | extracellular matrix protein 2, female organ |
| **Annotation Cluster 10** | **Enrichment Score: 3.07** |  |  |  | and adipocyte specific |
| **Gene Ontology ID** | **Gene Ontology Term** | ***P* value** |  | 214702_at, 214701_s_at | fibronectin 1 |
| UP_SEQ_FEATURE | region of interest:Triple-helical region | 0.05 |  | 203821_at | heparin-binding EGF-like growth factor |
| SP_PIR_KEYWORDS | hydroxylation | < .01 |  | 1555778_a_at, 210809_s_at | periostin, osteoblast specific factor |
| SP_PIR_KEYWORDS | triple helix | 0.01 |  | 206007_at | proteoglycan 4 |
| SP_PIR_KEYWORDS | hydroxylysine | 0.01 |  | 227480_at | sushi domain containing 2 |
| SP_PIR_KEYWORDS | hydroxyproline | 0.02 |  | 201108_s_at, 201107_s_at, 235086_at | thrombospondin 1 |
| GOTERM_CC_FAT | GO:0005581~collagen | 0.02 |  | 215646_s_at, 204619_s_at | versican |
| INTERPRO | IPR008160:Collagen triple helix repeat | 0.21 |  |  |  |
| SP_PIR_KEYWORDS | collagen | 0.07 |  |  |  |
|  |  |  |  |  |  |
| **Annotation Cluster 11** | **Enrichment Score: 3.02** |  |  |  |  |
| **Gene Ontology ID** | **Gene Ontology Term** | ***P* value** |  |  |  |
| INTERPRO | IPR013032:EGF-like region, conserved site | 0.07 |  |  |  |
| SP_PIR_KEYWORDS | EGF-like domain | 0.01 |  |  |  |
| UP_SEQ_FEATURE | domain:EGF-like 1 | 0.13 |  |  |  |
| INTERPRO | IPR000742:EGF-like, type 3 | 0.11 |  |  |  |
| INTERPRO | IPR006210:EGF-like | 0.12 |  |  |  |
| SMART | SM00181:EGF | 0.17 |  |  |  |
|  |  |  |  |  |  |
| **Annotation Cluster 12** | **Enrichment Score: 2.98** |  |  |  |  |
| **Gene Ontology ID** | **Gene Ontology Term** | ***P* value** |  |  |  |
| GO:0010941 | regulation of cell death | 0.04 |  |  |  |
| GO:0042981 | regulation of apoptosis | 0.07 |  |  |  |
| GO:0043067 | regulation of programmed cell death | 0.07 |  |  |  |
|  |  |  |  |  |  |
| **Annotation Cluster 13** | **Enrichment Score: 2.92** |  |  |  |  |
| **Gene Ontology ID** | **Gene Ontology Term** | ***P* value** |  |  |  |
| GO:0030036 | actin cytoskeleton organization | 0.03 |  |  |  |
| GO:0030029 | actin filament-based process | 0.04 |  |  |  |
| GO:0007010 | cytoskeleton organization | 0.34 |  |  |  |
|  |  |  |  |  |  |
| **Annotation Cluster 14** | **Enrichment Score: 2.83** |  |  |  |  |
| **Gene Ontology ID** | **Gene Ontology Term** | ***P* value** |  |  |  |
| UP_SEQ_FEATURE | domain:TSP C-terminal | 0.17 |  |  |  |
| UP_SEQ_FEATURE | repeat:TSP type-3 1 | 0.17 |  |  |  |
| UP_SEQ_FEATURE | repeat:TSP type-3 2 | 0.17 |  |  |  |
| UP_SEQ_FEATURE | repeat:TSP type-3 3 | 0.17 |  |  |  |
| UP_SEQ_FEATURE | repeat:TSP type-3 4 | 0.17 |  |  |  |
| UP_SEQ_FEATURE | repeat:TSP type-3 5 | 0.17 |  |  |  |
| UP_SEQ_FEATURE | repeat:TSP type-3 6 | 0.17 |  |  |  |
| UP_SEQ_FEATURE | repeat:TSP type-3 7 | 0.17 |  |  |  |
| UP_SEQ_FEATURE | repeat:TSP type-3 8 | 0.17 |  |  |  |
| INTERPRO | IPR008859:Thrombospondin, C-terminal | 0.13 |  |  |  |
| INTERPRO | IPR003367:Thrombospondin, type 3-like repeat | 0.13 |  |  |  |
| INTERPRO | IPR017897:Thrombospondin, type 3 repeat | 0.13 |  |  |  |
|  |  |  |  |  |  |
| **Annotation Cluster 15** | **Enrichment Score: 2.49** |  |  |  |  |
| **Gene Ontology ID** | **Gene Ontology Term** | ***P* value** |  |  |  |
| UP_SEQ_FEATURE | domain:TSP N-terminal | 0.26 |  |  |  |
| INTERPRO | IPR003129:Laminin G, thrombospondin-type, | 0.18 |  |  |  |
|  | N-terminal |  |  |  |  |
| SMART | SM00210:TSPN | 0.13 |  |  |  |
|  |  |  |  |  |  |
| **Annotation Cluster 16** | **Enrichment Score: 2.41** |  |  |  |  |
| **Gene Ontology ID** | **Gene Ontology Term** | ***P* value** |  |  |  |
| GO:0034329 | cell junction assembly | 0.10 |  |  |  |
| GO:0007044 | cell-substrate junction assembly | 0.14 |  |  |  |
| GO:0034330 | cell junction organization | 0.24 |  |  |  |
|  |  |  |  |  |  |
| **Annotation Cluster 17** | **Enrichment Score: 2.32** |  |  |  |  |
| **Gene Ontology ID** | **Gene Ontology Term** | ***P* value** |  |  |  |
| GO:0051271 | negative regulation of cell motion | 0.07 |  |  |  |
| GO:0030336 | negative regulation of cell migration | 0.24 |  |  |  |
| GO:0040013 | negative regulation of locomotion | 0.26 |  |  |  |
|  |  |  |  |  |  |
| **Annotation Cluster 18** | **Enrichment Score: 2.21** |  |  |  |  |
| **Gene Ontology ID** | **Gene Ontology Term** | ***P* value** |  |  |  |
| GO:0055010 | ventricular cardiac muscle morphogenesis | 0.13 |  |  |  |
| GO:0060415 | muscle tissue morphogenesis | 0.20 |  |  |  |
| GO:0055008 | cardiac muscle tissue morphogenesis | 0.20 |  |  |  |
| GO:0048844 | artery morphogenesis | 0.34 |  |  |  |
|  |  |  |  |  |  |
| **Annotation Cluster 19** | **Enrichment Score: 2.20** |  |  |  |  |
| **Gene Ontology ID** | **Gene Ontology Term** | ***P* value** |  |  |  |
| UP_SEQ_FEATURE | domain:EGF-like 1 | 0.13 |  |  |  |
| UP_SEQ_FEATURE | domain:EGF-like 4 | 0.40 |  |  |  |
| INTERPRO | IPR001881:EGF-like calcium-binding | 0.27 |  |  |  |
| INTERPRO | IPR018097:EGF-like calcium-binding, | 0.27 |  |  |  |
|  | conserved site |  |  |  |  |
| INTERPRO | IPR013091:EGF calcium-binding | 0.38 |  |  |  |
| SMART | SM00179:EGF_CA | 0.27 |  |  |  |
|  |  |  |  |  |  |
| **Annotation Cluster 20** | **Enrichment Score: 2.14** |  |  |  |  |
| **Gene Ontology ID** | **Gene Ontology Term** | ***P* value** |  |  |  |
| GO:0044449 | contractile fiber part | 0.06 |  |  |  |
| GO:0043292 | contractile fiber | 0.06 |  |  |  |
| GO:0030017 | sarcomere | 0.11 |  |  |  |
| GO:0030016 | myofibril | 0.15 |  |  |  |
|  |  |  |  |  |  |
| **Annotation Cluster 21** | **Enrichment Score: 2.11** |  |  |  |  |
| **Gene Ontology ID** | **Gene Ontology Term** | ***P* value** |  |  |  |
| UP_SEQ_FEATURE | domain:VWFC | 0.17 |  |  |  |
| INTERPRO | IPR001007:von Willebrand factor, type C | 0.37 |  |  |  |
| SMART | SM00214:VWC | 0.32 |  |  |  |
|  |  |  |  |  |  |
| **Annotation Cluster 22** | **Enrichment Score: 2.02** |  |  |  |  |
| **Gene Ontology ID** | **Gene Ontology Term** | ***P* value** |  |  |  |
| SP_PIR_KEYWORDS | ANK repeat | 0.04 |  |  |  |
| INTERPRO | IPR002110:Ankyrin | 0.19 |  |  |  |
| SMART | SM00248:ANK | 0.23 |  |  |  |
| UP_SEQ_FEATURE | repeat:ANK 1 | 0.69 |  |  |  |
| UP_SEQ_FEATURE | repeat:ANK 2 | 0.68 |  |  |  |
