# Supplementary material for: Human Subperitoneal Fibroblast and Cancer Cell Interaction Creates Microenvironment That Enhances Tumor Progression and Metastasis
Source: PLoS One. 2014 Feb 4;9(2):e88018. doi: 10.1371/journal.pone.0088018 (PMC3913740; doi:10.1371/journal.pone.0088018)
Supplement: Table S6 — Top 20 upregulated genes in SPFs with cancer cell-conditioned medium (CCCM) stimulation compared with SMFs with CCCM stimulation. (DOCX) [file pone.0088018.s008.docx]

| **Table S6. Top 20 upregulated genes in SPFs with cancer cell-conditioned medium (CCCM) stimulation compared with SMFs with CCCM stimulation** | | | | |
| --- | --- | --- | --- | --- |
| **Probe Set ID** | ***P* value** | **FC** | **Gene Symbol** | **Gene Title** |
| 229649_at | < .01 | 364.9 | NRXN3 | neurexin 3 |
| 200606_at | < .01 | 203.4 | DSP | desmoplakin |
| 204338_s_at | < .01 | 153.6 | RGS4 | regulator of G-protein signaling 4 |
| 205475_at | < .01 | 124.0 | SCRG1 | stimulator of chondrogenesis 1 |
| 206067_s_at | < .01 | 114.2 | WT1 | Wilms tumor 1 |
| 225728_at | < .01 | 93.0 | SORBS2 | sorbin and SH3 domain containing 2 |
| 203824_at | < .01 | 80.7 | TSPAN8 | tetraspanin 8 |
| 227006_at | < .01 | 70.3 | PPP1R14A | protein phosphatase 1, regulatory (inhibitor) subunit 14A |
| 204337_at | < .01 | 68.8 | RGS4 | regulator of G-protein signaling 4 |
| 206858_s_at | < .01 | 67.3 | HOXC6 | homeobox C6 |
| 213764_s_at | < .01 | 50.0 | MFAP5 | microfibrillar associated protein 5 |
| 219773_at | < .01 | 40.7 | NOX4 | NADPH oxidase 4 |
| 201596_x_at | < .01 | 39.6 | KRT18 | keratin 18 |
| 235337_at | < .01 | 38.4 | SERTAD4 | SERTA domain containing 4 |
| 226869_at | < .01 | 36.6 | MEGF6 | multiple EGF-like-domains 6 |
| 227337_at | < .01 | 33.6 | ANKRD37 | ankyrin repeat domain 37 |
| 202291_s_at | < .01 | 33.2 | MGP | matrix Gla protein |
| 206029_at | < .01 | 29.8 | ANKRD1 | ankyrin repeat domain 1 (cardiac muscle) |
| 205713_s_at | < .01 | 24.9 | COMP | cartilage oligomeric matrix protein |
| 209396_s_at | < .01 | 20.6 | CHI3L1 | chitinase 3-like 1 (cartilage glycoprotein-39) |
