# Supplementary material for: Human Subperitoneal Fibroblast and Cancer Cell Interaction Creates Microenvironment That Enhances Tumor Progression and Metastasis
Source: PLoS One. 2014 Feb 4;9(2):e88018. doi: 10.1371/journal.pone.0088018 (PMC3913740; doi:10.1371/journal.pone.0088018)
Supplement: Materials and Methods S1 — Supplementary Materials and Methods. (DOCX) [file pone.0088018.s009.docx]

**Supplementary Materials and Methods**

***Characteristics of Patients Entered into Area-Specific Tissue Microarrays***

One hundred and forty nine-patients with elastic laminal invasion (ELI) were entered into area-specific tissue microarrays (Table S1). They comprised 88 males and 61 females, with a mean age of 63.6 years. Of these, 67 had a tumor located in the right side, and 82, in the left side of the colon. TNM stages, using the UICC-TNM classification 5^th^ edition, were stage II in 49 patients, stage III in 53 patients, and stage IV in 47 patients. Curative resection was performed in 110 patients, and palliative resection was performed in 39. Lymph node metastasis was found in 57 patients, and was not found in 92. Patients were examined and 126 had well or moderately differentiated adenocarcinoma; 3 had mucinous adenocarcinoma, and 20 had poorly differentiated adenocarcinomas. Ninety-one tumors were diagnosed as pT3, and 58, as pT4.

***Antibodies and Reagents***

Antibodies used for immunohistochemical or immunocytochemical stainings were monoclonal mouse anti-human CD3 (UCHT1), CD31 (JC70A), CD68 (PG-M1), α-smooth muscle actin (α-SMA, 1A4), vimentin (Vim 3B4), and polyclonal rabbit anti-S100, from DakoCytomation (Glostrup, Denmark). For flow cytometry analysis, fluorochrome-conjugated mouse anti-human CD14 (61D3), CD20 (2H7), and CD45 (HI30) were purchased from eBioscience (San Diego, CA). Fluorochrome-conjugated and unconjugated mouse anti-human CD3 (UCHT1), CD34 (QBEnd10), CD68 (KP1), CD105 (SN6h), and CD117 were purchased from DakoCytomation (Glostrup, Denmark). Unconjugated mouse anti-human CD133 (AC133) was purchased from Miltenyi Biotec (Glabach,Germany). Fluorochrome-conjugated forms of mouse IgG1κ, IgG2bκ, and rat IgG2bκ isotype controls were purchased from eBioscience (San Diego, CA). The information is summarized in Supplementary Table S2.

***Immunohistochemical staining***

Immunohistochemical staining was performed using an autostainer (Ventana Benchmark^®^, Roche Diagnostics, Tokyo, Japan). Paraffin-embedded 5-μm tissue microarray sections were incubated with primary antibodies for 1 hour. Diaminobenzidine was used as a chromogen and slides were counterstained with hematoxylin before mounting.

***Cell Proliferation Assay***

A total of 3 sets of SMFs and SPFs from 3 different patients were used for cell proliferation assays. Cells (5 x 10^4^) were placed into a 6-well plate and cell counts were performed by day 4. Cells were trypsinized, centrifuged, and stained with Tripan blue. Viable cells were counted using a hemocytometer. Doubling time (D.T) was calculated with the following formula:

D.T = 96 x log(2) / log(cell number at day 4) - log(cell number at day0).

***Immunocytochemical Staining***

For the characterization of SMFs and SPFs, cells were plated at a density of 1.7 x 10^4^ cells/cm^2^ and cultured until semi-confluent on a culture slide (Becton Dickinson Labware, Franklin Lake, NJ). Cultured cells were fixed in cold 4% paraformaldehyde for 15minutes on ice. After fixation, blocking was performed in 5% milk in phosphate buffered saline (PBS) for 30 minutes. Cells were then incubated with primary antibodies at room temperature for 1 hour. After they were washed with PBS, the slides were incubated with EnVision (DAKO, Glostrup, Denmark) for 1hour at room temperature. After they were washed again with PBS, each slide was incubated for 5 minutes in 2% 3,3’-diaminobenzidine tetrahydrochloride in 50mM Tris-buffer (pH7.6) containing 0.3% hydrogen peroxidase as a chromogen, and then counters-tained with hematoxylin. As positive controls, formalin-fixed and paraffin-embedded human peripheral nerve tissue, mesothelial tissue, and smooth muscle tissue were used.

***Flow cytometry analysis***

Flow cytometry was performed to confirm the immunohistochemical features consistent with fibroblasts in the obtained primary cells. FACSCalibur cytometer (Beckton-Dickinson, San Jose, CA) was used and a minimum of 10,000 events were counted with Cell Quest software (Beckton-Dickinson Labware, NJ). Cells were plated at a density of 1.7 x 10^4^ cells/cm^2^ and cultured until semiconfluent on 10 cm slides. Cells were trypsinized, centrifuged, and incubated with primary antibodies for 15 minutes on ice, in the dark. Excess antibodies were removed by washing with PBS containing 3% FBS and 0.05% NaN_3_. Next, in the case of unconjugated primary antibodies, secondary antibodies (FITC-conjugated polyclonal rabbit anti-mouse and polyclonal mouse anti-rabbit immunoglobulins (DakoCytomation, Glostrup, Denmark) were added and incubated for 15 minutes on ice, in the dark. The cells were washed twice with PBS and a FACS scan was performed.
